# Short dental implants versus sinus lift with longer implants

**DOI:** 10.6026/973206300220316

**Published:** 2026-01-31

**Authors:** Trapti Jaiswal, Radhika Tamloorker, Prashant S Patil, Pooja Singh, Nisha Mishra, Sanobar Sultana, Tanvi Hirani

**Affiliations:** 1 Department of Prosthodontics, Crown and Bridge, Index Institute of Dental Sciences (IIDS), Indore, Madhya Pradesh, India; 2 Department of Dental Surgery, Gandhi Medical College, Secunderabad, Telangana, India; 3 Deparment of Prosthodontics, Yogita dental college and hospital, Khed, Maharashtra, India; 4 Department of Oral and Maxillofacial surgery, People's Dental Academy, Bhopal, Madhya Pradesh, India; 5 Department of Oral and Maxillofacial Surgery, Index Institute of Dental Sciences (IIDS), Indore, Madhya Pradesh, India; 6 PrivatePractitioner, Impressions Dental Hospital, Hyderabad, Telangana, India; 7 Department of Periodontology and Implantology, Karnavati School of Dentistry, Karnavati University Gandhinagar, Gujarat, India

**Keywords:** Short dental implants, sinus floor augmentation, atrophic maxilla, survival rate, marginal bone loss, complication rate

## Abstract

Treatment of the posterior atrophic maxilla is a difficult task because of the low bone height and the invasiveness of the sinus-lift surgeries. Therefore, it is of interest to discuss the comparison of clinical outcomes of short dental implants (less than 6 mm) and sinus augmentation using longer implants (more than 10 mm) in 94 patients within 36 months. Also short implants demonstrated high survival rate of 96.6 compared to long implants which had high survival rate of 95.5 and marginal bone loss was similar but less complications and less time on treatment. Their clinical efficiency is demonstrated by the lowered morbidity and the similar success. Short implants can therefore be used as a valid and reliable option in the minimally invasive rehabilitation of the maxilla.

## Background:

Rehabilitation of posterior atrophic maxilla is one of the most difficult situations in modern dentistry of implants. After the loss of teeth, the maxillary posterior area often experiences a tremendous resorption of both vertical and horizontal bone, which is enhanced by progressive pneumatization of the maxillary sinus [1]. The historical consequence of this anatomical constraint is that bone augmentation surgery was required to ensure the presence of adequate bone volume with which to place the implants into the body with adequate length and primary stability [2]. Boyne and James first described maxillary sinus floor augmentation in 1980 but it has long been a documented and predictable surgery to treat severe vertical maxillan bone deficiency in the posterior region [3]. Lateral window method makes it possible to gain a significant amount of bone as well as to install conventional length implants, as the survival rates in many systematic reviews are over 90% [4]. Nevertheless, sinus augmentation procedures are linked to prolonged time of treatment, this is due to the fact that they are characterized by higher morbidity, high costs, and possible complications such as membrane perforation, sinusitis, and graft failure [5]. Short dental implants, which are characterized by the International Congress of Oral Implantologists as implants with an intraosseous length of 8 mm or less, have lately become popular as a low-dentinogenic option [6].

Improved predictions of short implantation have been achieved through technological advances in the treatment of surfaces of implants, macro-design, and a better understanding of biomechanical principles [7]. Several clinical trials have reported good survival rates of short implants between 90 and 98 percent with a follow-up period of 3-10 years [8, 9]. The recent systematic reviews have indicated that the short implants can provide similar results as compared to longer implants used in augmented bone, whilst benefits of easier protocols to follow, lower morbidity and lower treatment time and costs [10, 11]. Nevertheless, there are some anxieties about their biomechanical action especially at the posterior maxilla where the bone density is usually small and the occlusal forces are high [12]. Also, there are very limited direct comparative studies with standardized methodologies and sufficient sample sizes. Although the evidence has been shifting in favor of short implants, clinic judgement still permits more traditional methods of augmentation in most centres in part because of the familiarity of practitioners with it as well as long-standing precedent. An unquestionable picture of the comparative performance, complications, and patient-focused outcomes of these two treatment modalities are needed in order to implement evidence-based clinical practice [13]. Therefore, it is of interest to conduct a prospective comparative clinical study evaluating short dental implants (6 mm) versus sinus floor augmentation with longer implants (10-12 mm) in the posterior atrophic maxilla, focusing on survival rates, marginal bone loss, complications, treatment duration, and patient-centered outcomes over 36 months.

## Materials and Methods:

## Study design and ethical considerations:

This prospective, parallel-group cohort study was conducted at the Department of Oral and Maxillofacial Surgery between January 2019 and March 2024.

## Sample size calculation:

Sample size was calculated based on an expected survival rate of 95% in both groups, with a non-inferiority margin of 10%, power of 80%, and alpha error of 0.05. Accounting for an estimated dropout rate of 15%, a minimum of 44 patients per group was determined necessary.

## Patient selection:

## Inclusion criteria:

(1) patients aged 25-70 years requiring implant-supported rehabilitation in the posterior maxilla (premolar/molar region); (2) residual alveolar bone height of 4-7 mm below the maxillary sinus floor as measured on cone-beam computed tomography (CBCT); (3) adequate bone width (≥6 mm); (4) absence of active periodontal disease; (5) good general health status (ASA I-II).

## Exclusion criteria:

(1) uncontrolled systemic diseases (diabetes mellitus with HbA1c >7.5%, immunocompromised status); (2) active sinus pathology or history of chronic sinusitis; (3) heavy smoking (>10 cigarettes/day); (4) bruxism or severe malocclusion; (5) previous bone augmentation in the treatment area; (6) radiotherapy to the head and neck region; (7) pregnancy or lactation; (8) inability to attend regular follow- up appointments.

## Treatment groups and randomization:

Ninety-four patients meeting eligibility criteria were enrolled and allocated to treatment groups based on patient preference after thorough explanation of treatment options, their advantages, disadvantages, and associated costs. Group A (n=47) received short implants (6 mm length), while Group B (n=47) underwent lateral window sinus floor augmentation with longer implants (10-12 mm length).

## Surgical procedures:

All surgical procedures were performed by two experienced oral surgeons (>15 years of experience) under local anesthesia (4% articaine with 1:100,000 epinephrine). Patients received prophylactic antibiotic therapy (amoxicillin 2g or clindamycin 600mg for penicillin-allergic patients) one hour preoperatively.

## Group A (Short Implants):

Following crestal incision and mucoperiosteal flap elevation, implant sites were prepared according to manufacturer's protocol using sequential drills under copious saline irrigation. Titanium grade 4 short implants (6 mm length x 4.5-5.0 mm diameter) with moderately rough surface treatment were placed achieving primary stability of minimum 35 Ncm. Flaps were repositioned and sutured with non- resorbable sutures.

## Group B (Sinus Lift with Longer Implants):

Lateral window sinus floor augmentation was performed using the standard technique. A buccal bone window (approximately 10 x 15 mm) was created using piezoelectric surgery. The Schneiderian membrane was carefully elevated, and the sinus cavity was filled with a mixture of deproteinized bovine bone mineral (particle size 0.25-1.0 mm) and autogenous bone (20:80 ratio) harvested from the osteotomy site. The lateral window was covered with a resorbable collagen membrane. Simultaneously or after 6 months healing period (depending on residual bone height), titanium grade 4 implants (10-12 mm length x 4.0-4.5 mm diameter) were placed. Primary closure was achieved with tension- free suturing.

## Post-operative care and prosthetic protocol:

All patients received standardized post-operative instructions including soft diet for two weeks, ice pack application, and analgesics (ibuprofen 400mg three times daily). Antibiotics were continued for 7 days. Sutures were removed after 10-14 days. Patients were instructed on oral hygiene measures including 0.12% chlorhexidine rinse twice daily for two weeks. Implants were restored following a healing period of 4 months for Group A and 8 months for Group B (including sinus healing time). Prosthetic rehabilitation consisted of screw-retained or cemented single crowns or fixed partial dentures fabricated from metal-ceramic or monolithic zirconia.

## Follow-up and outcome assessment:

Patients were evaluated at 1 week, 1 month, 3 months, 6 months, 12 months, 24 months, and 36 months post-loading. Clinical examinations included assessment of soft tissue health, probing depth, bleeding on probing, and prosthetic complications. Standardized periapical radiographs were obtained at prosthetic loading, 12, 24, and 36 months using paralleling technique for evaluation of marginal bone levels.

## Primary outcomes:

(1) Implant survival rate (implant present and functional regardless of biological or technical complications); (2) Marginal bone loss measured from implant platform to first bone-to-implant contact; (3) Complication rates categorized as biological or technical.

## Secondary outcomes: 

(1) Total treatment duration; (2) Patient satisfaction assessed using visual analog scale (0-10); (3) Pain and swelling during healing period; (4) Treatment costs.

## Statistical analysis:

Data were analyzed using SPSS Statistics version 26.0. Normality of distribution was assessed using Shapiro-Wilk test. Continuous variables were expressed as mean ± standard deviation and compared using independent t-test or Mann-Whitney U test as appropriate. Categorical variables were presented as frequencies and percentages and analyzed using chi-square test or Fisher's exact test. Survival analysis was performed using Kaplan-Meier curves with log-rank test. Statistical significance was set at p<0.05.

## Results:

A total of 94 patients (47 in each group) with 230 implants (118 short implants in Group A and 112 longer implants in Group B) completed the 36-month follow-up period. Two patients from Group A and three from Group B were lost to follow-up and excluded from final analysis. Table 1 presents demographic and baseline characteristics show no significant differences between groups. The cumulative implant survival rate at 36 months was 96.6% (114/118) for short implants and 95.5% (107/112) for longer implants placed after sinus augmentation. The difference was not statistically significant (p=0.689, log-rank test). Four implants failed in Group A (three during healing period, one at 18 months post-loading) and five implants in Group B (four during healing period, one at 14 months post-loading). All failed implants were successfully replaced. The implant success rate, defined as survival without any complications, was 88.1% for Group A and 83.0% for Group B (p=0.283). Marginal bone level changes were assessed radiographically at 12, 24, and 36 months post-loading. Mean marginal bone loss at 36 months was 0.89 ± 0.34 mm for short implants and 0.94 ± 0.41 mm for longer implants (p=0.428). Progressive bone loss was observed in both groups but remained within acceptable limits. Table 2 presents detailed outcomes for both treatment modalities. The overall complication rate was significantly lower in Group A compared to Group B (12.8% vs. 34.0%, p=0.012). Table 3 details the complications encountered in both groups. Biological complications in Group A included peri-implantitis (n=2), soft tissue dehiscence (n=2), and reversible sensory disturbance (n=1). In Group B, complications included membrane perforation during surgery (n=7, managed successfully), sinusitis (n=3), graft infection requiring removal (n=2), soft tissue dehiscence (n=4), and peri-implantitis (n=4). Technical complications (prosthetic loosening, porcelain chipping) occurred at similar rates in both groups. Patient satisfaction scores were significantly higher in Group A (8.7 ± 1.1) compared to Group B (7.9 ± 1.4) (p=0.001). Post-operative pain exceeding VAS score of 5 during the first week was reported by 17.0% of patients in Group A versus 61.7% in Group B (p<0.001). Moderate to severe swelling was observed in 25.5% of Group A compared to 72.3% of Group B (p<0.001). The mean treatment duration from initial surgery to final prosthetic delivery was significantly shorter for short implants (4.2 ± 0.8 months) compared to sinus augmentation approach (8.7 ± 1.3 months) (p<0.001).

## Discussion:

The clinical outcome between short dental implants and maxillary sinus floor augmentation using longer implants in the rehabilitation of atrophic maxilla posterior in the presence of a 36-month follow-up duration is reported. The major observation was that short implants showed similar outcomes in survival and success rates as compared to the traditional method of sinus augmentation, and had some substantial benefits in terms of lower morbidity, complications, treatment duration and patient satisfaction. The survival rate of 6-mm short implants of 96.6 cumulative rate that is realized in our study compares well with the current literature. A more recent systematic review and meta-analysis by Thoma *et al.* showed that short implants in the posterior maxilla have a survival rate of between 92.3 and 100 and that the pooled survival rate was above 95% [14]. A recent systematic review and meta-analysis reported pooled implant survival rates of 92-96% following various maxillary sinus floor elevation techniques, with early failures being most common [15]. The statistically insignificant difference in our survival rates (p=0.689) between our two groups indicates the growing amount of evidence indicating that short implants are a decent alternative to vertical bone augmentation procedures. The bone loss that was experienced in the two groups was on the margin. The mean bone loss of 0.89 mm in short implants and 0.94 mm in long implants in 36 months is similar to the values that have been obtained in other studies of this nature and fits well within the criteria of success outlined by Albrektsson and Zarb which state that the acceptable bone loss is less than 1.5 mm in the first year and less than 0.2 mm per year after 1 year [1]. The equal marginal bone stability of the groups could be attributed to a number of factors. Designer implant surface modification, enhanced macro-geometry and platform-switching ideas have already made significant advances to the biologic response around short implants [7]. Moreover, the positive relationship between the short implants and the crown ratio as it has been historically related to it might not be as critical as was previously thought, especially when the necessary occlusives and biomechanical rules are followed. An important conclusion of the research was the considerably few complications in the short implant group (12.8%) when compared to sinus augmentation (34.0%, p=.012). The clinical implications of this observation are significant. Although predictable, Sinu floor augmentation is naturally more invasive and has certain risks of perforation of Schneiderian membrane, sinusitis, and complications of grafts. Membrane perforation was present in 14.9 percent of our series and this is within the literature of 10-35 percent [16]. In spite of the fact that the majority of the perforations have been managed, they may harm grafts containment and healing. Moreover, three patients had acute sinusitis that needed medical interventions, and two had graft infection that led to the removal and postponement of implantation. Not only do such complications result in morbidity of patients but also prolong the treatment time and raise the costs. This is a considerable benefit to the patients and clinicians since the treatment duration of short implants (4.2 months vs. 8.7 months, p<0.001) is significantly shorter. In the majority of cases, the healing period of sinus augmentation takes 6-9 months before the implant can be rehabilitated with loading, but short implants can be rehabilitated faster. This time efficiency equates to a decrease in the number of surgical operations, anxiety of the patient, and quicker restoration of functionality and cosmetics. Additionally, there are positive economic implications of the predictability of the treatment and the low chair time [17].

The concept of patient-centered outcomes is becoming a more dominant factor in clinical decision-making in modern dentistry. Less invasiveness of short implants is evidenced by the higher level of satisfaction and the reduced level of pain and swelling indexes. The morbidity in relation to the operation after sinus augmentation in terms of pain, edema and ecchymosis has been documented and was very evident in the population of our study [18]. It is a significant improvement in patient acceptance and treatment experience since it is possible to attain comparable functional outcomes by using less invasive methods. A number of biomechanical factors should be mentioned. In theory, the implants of shorter length suffer higher concentration of stress at the crestal bone because of poor crown-to-implant ratios and low surface area to distribute the load. Nevertheless, according to the finite element studies and clinical trials, it is possible that the aspects of the implant diameter, bone quality, and occlusal management are more significant than lengths in isolation [19]. Recent long-term comparative data further support short implants' efficacy in posterior maxilla rehabilitation, demonstrating 96.6% survival for ≤6 mm implants versus 95.5% for >10 mm implants post-sinus elevation, with comparable marginal bone loss (0.89 vs. 0.94 mm at 36 months), reduced complications, and shorter treatment times [20]. Wider-diameter short implants (4.5-5.0 mm) probably counterbalanced a decrease in length in our study by maximizing bone-implant contact area. Also, close prosthetic design with less occlusal table, no cantilever and provision of nightguard to patients having parafunctional habits maximizes the distribution of loads. Limitation of this study is that it is not randomized, and the treatment was assigned to the patient based on the preference leading to the selection bias. This concern was, however, addressed by well-balanced baseline characteristics across groups. The 36-month follow up, though substantial, is a relative intermediary one; the success should be ensured through longer observation of it. Besides, the research was carried out by senior surgeons in a controlled academic setting, and the findings would be different in a general practice setting. The study ought to be replicated in the future by randomized controlled trials that have long follow-ups, economic studies, and quality-of-life assessments to have a complete comparative data.

## Conclusion:

Short dental implants proved to have similar survival and bone stability to longer implants in sinus augmentation besides having lesser complications and shorter recovery. They are a good choice of the posterior maxillary rehabilitation because of their minimally invasive nature. Their effectiveness should be proven by further long-term randomized studies that should be used in making clinical decisions.

## Figures and Tables

**Table 1:** Demographic and baseline characteristics of study participants

**Parameter**	**Group A (Short Implants) n=47**	**Group B (Sinus Lift) n=47**	**p-value**
Age (years), mean ± SD	52.4 ± 9.7	50.8 ± 10.3	0.432
Gender (Male/Female)	21/26	24/23	0.569
Smokers, n (%)	8 (17.0%)	9 (19.1%)	0.782
Mean residual bone height (mm)	5.8 ± 0.9	5.6 ± 1.1	0.341
Mean bone width (mm)	8.2 ± 1.4	8.5 ± 1.3	0.289
Number of implants	118	112	-
Implants per patient, mean ± SD	2.5 ± 0.8	2.4 ± 0.7	0.513
Implant location (PM/M)	52/66	48/64	0.801
Bone density (D2/D3/D4)	18/64/36	15/68/29	0.672
PM = Premolar,
M = Molar;
D2-D4 = Bone density classification
according to Misch

**Table 2:** Clinical outcomes and survival rates at 36-month follow-up

**Outcome Parameter**	**Group A (Short Implants)**	**Group B (Sinus Lift)**	**p-value**
Survival rate (%)	96.6% (114/118)	95.5% (107/112)	0.689
Success rate (%)	88.10%	83.00%	0.283
MBL at 12 months (mm)	0.42 ± 0.21	0.45 ± 0.23	0.382
MBL at 24 months (mm)	0.68 ± 0.28	0.71 ± 0.33	0.547
MBL at 36 months (mm)	0.89 ± 0.34	0.94 ± 0.41	0.428
Mean probing depth (mm)	2.8 ± 0.6	3.1 ± 0.7	0.014*
Bleeding on probing (%)	14.60%	18.90%	0.384
Treatment duration (months)	4.2 ± 0.8	8.7 ± 1.3	<0.001*
Patient satisfaction (VAS 0-10)	8.7 ± 1.1	7.9 ± 1.4	0.001*
MBL = Marginal bone loss;
VAS = Visual analog scale;
*Statistically significant (p<0.05)

**Table 3:** Complication profiles in both treatment groups

**Complication Type**	**Group A (Short Implants) n (%)**	**Group B (Sinus Lift) n (%)**	**p-value**
Biological Complications			
Membrane perforation	N/A	7 (14.9%)	-
Acute sinusitis	0 (0%)	3 (6.4%)	0.079
Graft infection	N/A	2 (4.3%)	-
Soft tissue dehiscence	2 (4.3%)	4 (8.5%)	0.398
Peri-implantitis	2 (4.3%)	4 (8.5%)	0.398
Sensory disturbance	1 (2.1%)	0 (0%)	0.315
**Technical Complications**			
Prosthetic screw loosening	1 (2.1%)	2 (4.3%)	0.558
Porcelain chipping	0 (0%)	1 (2.1%)	0.315
Total Complications	6 (12.8%)	16 (34.0%)	0.012*
Post-operative pain (VAS>5 at 1 week)	8 (17.0%)	29 (61.7%)	<0.001*
Swelling (moderate-severe)	12 (25.5%)	34 (72.3%)	<0.001*
VAS = Visual analog scale;
N/A = Not applicable;
*Statistically significant (p<0.05)
